# Genome Sequences of SARS-CoV-2 Strains Detected in Hong Kong

**DOI:** 10.1128/MRA.00697-20

**Published:** 2020-07-30

**Authors:** Chun Hang Au, Wai Sing Chan, Ho Yin Lam, Dona N. Ho, Simon Y. M. Lam, Jonpaul S. T. Zee, Tsun Leung Chan, Edmond S. K. Ma

**Affiliations:** a Molecular Pathology Division, Department of Pathology, Hong Kong Sanatorium & Hospital, Hong Kong; Queens College

## Abstract

We sequenced severe acute respiratory syndrome coronavirus 2 (SARS-CoV-2) genomes from deep throat saliva samples of three imported cases in Hong Kong by Nanopore sequencing. Epidemiological and clinical features of these coronavirus disease 2019 (COVID-19) cases were presented for genomic epidemiology studies.

## ANNOUNCEMENT

Severe acute respiratory syndrome coronavirus 2 (SARS-CoV-2; family *Coronaviridae* and genus *Betacoronavirus*) is causing an ongoing coronavirus disease 2019 (COVID-19) pandemic. There were 1,109 COVID-19 cases in Hong Kong as of 12 June 2020 (https://www.chp.gov.hk/files/pdf/local_situation_covid19_en.pdf), with most cases reported in March to April 2020. We report here the genome sequences of SARS-CoV-2 strains detected from three imported cases in Hong Kong.

Case 1 (58-year-old man, HKSH0003) and case 2 (61-year-old female, HKSH0004) are a couple who had symptom onset on 10 March 2020 after returning from the United States to Hong Kong and tested positive on 14 March. Case 1 was relatively asymptomatic apart from fever, but case 2 showed symptoms and signs of pneumonia during hospitalization. They were enrolled in a clinical trial of interferon beta-1b, lopinavir-ritonavir, and ribavirin (1) and were discharged after two negative PCR tests. Case 3 (59-year-old man, HKSH0007) had symptom onset on 21 March after returning from Germany to Hong Kong and tested positive on 1 April. He showed very mild symptoms and denied treatment during isolation. The patient was discharged after two negative PCR tests. Informed consent was obtained from every case. This study was reviewed and approved by the Research Committee of Hong Kong Sanatorium & Hospital (reference number RC-2020-11).

Deep throat saliva samples treated with Sputasol (Thermo Scientific, USA) were subjected to nucleic acid extraction using eMAG (bioMérieux, France). The full genome was amplified following the ARTIC nCoV-2019 V1 protocol (https://www.protocols.io/view/ncov-2019-sequencing-protocol-bbmuik6w). Briefly, cDNA was synthesized using a SuperScript III first-strand synthesis system (Invitrogen, USA) with random hexamers. Two multiplex PCRs of 49 amplicons (V1 panel) were performed using AmpliTaq Gold DNA polymerase (Applied Biosystems, USA), combined, purified using AMPure XP beads (Beckman Coulter, USA), and quantified using a Qubit double-stranded DNA (dsDNA) high-sensitivity assay (Invitrogen, USA). Each genome was sequenced separately. For each sample, a separate Nanopore sequencing library was prepared using a ligation sequencing kit (SQK-LSK109; Oxford Nanopore Technologies, UK), sequenced using a new MinION R9.4.1 flow cell (FLO-MIN106D) with MinKNOW live base calling version 19.12.2 (fast model), and analyzed following the ARTIC nCoV-2019 bioinformatics protocol. Briefly, filtered reads (mean quality score, ≥7; length, 400 to 700 bp) were mapped to the Wuhan-Hu-1 reference strain (GenBank accession number MN908947.3) using Minimap2 version 2.17 (parameter -x map-ont) and subjected to primer soft-clipping using align_trim (version 1.1.0) (https://artic.network/ncov-2019/ncov2019-bioinformatics-sop.html) or BAMClipper version 1.1.1 (parameter -u 40 -d 40) (2). Consensus genomes were built from Nanopolish version 0.12.5 (3) variant calls. Maximum-likelihood time-resolved phylogenetic analysis was conducted using TreeTime (4) and Nextstrain (5) (https://github.com/nextstrain/ncov) on 12 June 2020 (default GTR substitution model and nucleotide substitution rate of 0.0008 per site per year). Default parameters were used for all software unless otherwise specified.

**FIG 1:**
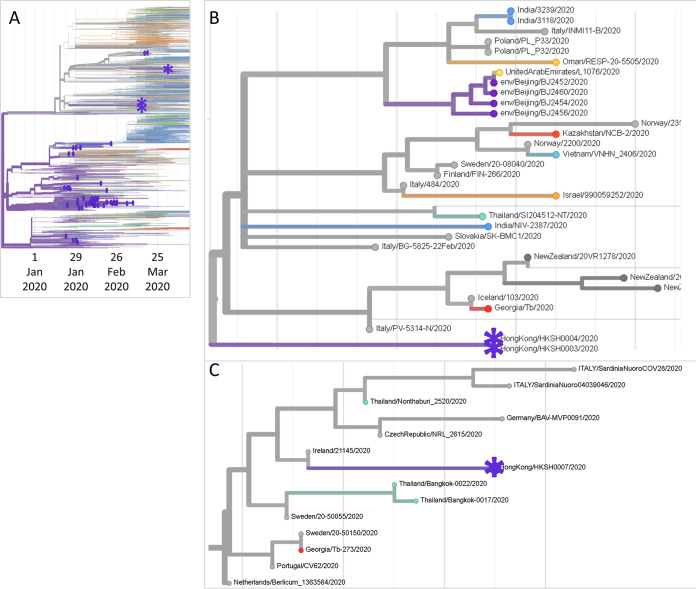
(A) Maximum-likelihood time-resolved phylogenetic tree of 2,949 SARS-CoV-2 genome sequences sampled up to 16 April 2020 in the GISAID database on 12 June 2020 (https://nextstrain.org/ncov/global?dmax=2020-04-16). Genome sequences sampled in Hong Kong are indicated as purple asterisks for the three sequences reported in this announcement and purple circles for the other 72 sequences. Phylogeny was rooted relative to early sequences from Wuhan, China. All tree branches are scaled according to sampling times (terminal nodes) and the most likely time of divergence (internal nodes). (B) Close-up view of the phylogeny (https://nextstrain.org/ncov/asia?dmax=2020-04-16) of case 1 (HKSH0003, purple asterisk), case 2 (HKSH0004, purple asterisk), and 29 closely related sequences (gray for non-Asian samples and other colors for Asian samples) (late February 2020 to mid-April 2020). (C) Close-up view of the phylogeny (https://nextstrain.org/ncov/asia?dmax=2020-04-16) of case 3 (HKSH0007, purple asterisk) and 13 closely related sequences (gray for non-Asian samples and other colors for Asian samples) (late February 2020 to mid-April 2020).

Nanopore sequencing runs yielded 0.497-Gbp reads for HKSH0003 (853,213 reads; mean length, 582 bp), 0.534-Gbp reads for HKSH0004 (781,048 reads; mean length, 684 bp), and 7.740-Gbp reads for HKSH0007 (16,122,660 reads; mean length, 480 bp). All three SARS-CoV-2 genomes were sequenced at a mean depth of >3,500×, reference coverage of 92% to 96%, length of 27,384 to 28,723 nucleotides, and GC content of 38%. Phylogenetic analysis showed that they descended from European samples (88%, 88%, and 100% confidence for cases 1, 2, and 3, respectively) and were distinct from most other Hong Kong sequences (Fig. 1A). Although the disease severities of cases 1 and 2 were different, there was no detectable difference between their SARS-CoV-2 genomes (Fig. 1B). This suggests that host factors played a more important role in clinical outcome than viral genetic variation, as shown by emerging clinical studies (6). The phylogeny of genomes related to case 3 was also consistent with a pattern of international dissemination by air travel (Fig. 1C).

### Data availability.

These sequences were deposited in the Global Initiative on Sharing All Influenza Data (GISAID) database (strain identifiers EPI_ISL_430018, EPI_ISL_430063, and EPI_ISL_451957) and in NCBI GenBank under the accession numbers MT510744, MT510745, and MT517419, BioProject number PRJNA634965, BioSample numbers SAMN15013847, SAMN15013848, and SAMN15028143, and SRA numbers SRR11844878, SRR11844877, and SRR11852109.
